# The Parameters Affecting Antimicrobial Efficiency of Antimicrobial Blue Light Therapy: A Review and Prospect

**DOI:** 10.3390/biomedicines11041197

**Published:** 2023-04-18

**Authors:** Shijie Huang, Shangfei Lin, Haokuan Qin, Hui Jiang, Muqing Liu

**Affiliations:** 1 School of Information Science and Technology, Fudan University, 2005th Songhu Road, Shanghai 200438, China; 2 Academy for Engineering and Technology, Fudan University, 220th Handan Road, Shanghai 200433, China; 3 Zhongshan Fudan Joint Innovation Center, 6th Xiangxing Road, Zhongshan 528403, China

**Keywords:** antimicrobial blue light, energy conversion, photochemical effect, photophysical effect, pulsed blue light

## Abstract

Antimicrobial blue light (aBL) therapy is a novel non-antibiotic antimicrobial approach which works by generating reactive oxygen species. It has shown excellent antimicrobial ability to various microbial pathogens in many studies. However, due to the variability of aBL parameters (e.g., wavelength, dose), there are differences in the antimicrobial effect across different studies, which makes it difficult to form treatment plans for clinical and industrial application. In this review, we summarize research on aBL from the last six years to provide suggestions for clinical and industrial settings. Furthermore, we discuss the damage mechanism and protection mechanism of aBL therapy, and provide a prospect about valuable research fields related to aBL therapy.

## 1. Introduction

Antibiotics have been used against pathogenic bacteria for a long time and there is no doubt that antibiotics have contributed to the control of infectious diseases significantly. However, the increasing occurrence of antibiotic-resistant pathogens, which have been attributed to the extensive use of antibiotics, greatly threatens human health and the ecological environment [1,2]. Studies have shown that bacteria can resist the action of antibiotics by modification of targets and drugs, drug efflux or establishing protective barriers [3]. The development of new drugs and the use of drug cocktails improve therapy to some extent, but the risks posed by antibiotic resistance genes (ARGs) that occur in the environment [4] still exist. The investigation of novel non-antibiotic approaches has been a focus of research in recent years, and antimicrobial blue light (aBL) therapy is one of these approaches. Antimicrobial blue light (in the wavelength of 400–480 nm) has shown antimicrobial properties to various bacteria pathogens [5,6]. A common hypothesis is that the antibacterial ability of aBL is due to endogenous photosensitizing chromophores of bacteria pathogens [7,8]. Endogenous photosensitizers (such as porphyrins and flavins) absorb photons of blue light, then generate reactive oxygen species (ROS) [7,9] which lead to oxidative damage of biomolecules (e.g., protein, DNA, lipid) and a decrease in viable bacteria [10,11]. It seems that bacteria pathogens due not easily form blue light resistance [12,13] due to the multi-target properties of aBL therapy.

Recent studies have shown that aBL is a potential method for disinfection (e.g., in the food industry [14,15]) and the treatment of localized infections (e.g., dental infections, acne, and cutaneous fungal infection) [16]. As a non-thermal technology, blue light showed a similar inactivation of *E. coli* in milk compared with pasteurization, with a non-significant change in the physico-chemical properties of milk [17]. As for the clinical situation, some researchers found that aBL might increase the shelf-life of platelets and other blood products [18,19]. Dai et al. and their colleagues used aBL for treating burns and wounds in infected mice successfully [12,20,21]. There are also some studies on aBL therapy for bacterial elimination in periodontal therapy in vivo and in vitro [22,23,24].

A lot of experiments have been designed to evaluate the antimicrobial efficiency of aBL, and a few review articles have tried to establish the relationship between bacteria survival and aBL parameters [25,26]. However, aBL therapy involves many parameters, which makes it difficult to summarize the relevant rules. In this review, we summarize studies on aBL therapy published in the last six years to provide suggestions for clinical and industrial settings. Furthermore, we discuss the damage mechanism and protection mechanism of aBL therapy, and describe the prospect of valuable research fields related to aBL therapy.

## 2. ABL Parameters Impact Antimicrobial Efficacy

We searched articles from the last six years (January 2017–December 2022) in a database (Web of Science), with key words associated with blue light and bacteria. We screened the titles and abstracts from 983 related records, and unrelated articles were excluded (e.g., photodynamic therapy, in vivo research). Finally, we selected 21 articles and sorted them into Table 1, Table 2 and Table 3. The light parameters of aBL are listed in Table 4.

The doses $D_{90}$ and $D_{99}$ were taken as characteristic value of the photobiological effect, which were required for inactivation of 90% and 99% of bacterial cells [8]. Since the doses $D_{90}$ and $D_{99}$ were not given in some studies, we estimated the values from the curve of bacteria survival and light dose.

### 2.1. Wavelength

In the spectrum of blue light (400–480 nm), the shorter wavelength light seems to show a better antibacterial effect in various experiments. A study showed that the $D_{90}$ of 405 nm and 445 nm are 99 J/cm^2^ and 609 J/cm^2^ in the treatment of *E. coli*, respectively [8]. Another study showed that 420 nm blue light achieved reductions up to 99%, whereas 455 nm blue light and 480 nm blue light were less effective (60–83%) [27]. A review reported that 470 nm is less effective by a factor of between 2 and 5 compared to 405 nm illumination [28].

The sensitivity of bacteria to aBL is different in various species and strains. Among those drug-resistant *E. coli*, the CTX-M-producing strain ST131 was the most susceptible to 410 nm blue light while the KPC-2-producing *E. coli* ST648 was the most tolerant strain when compared to all other tested strains [29]. Another study also indicated strain-dependent responses to 455 nm blue light [30].

Since the absorb spectrums of those endogenous photosensitizers are different and the energy carried by photons is different at different wavelengths, this will impact the photosensitization process (production of ROS) and lead to different photoinactivation efficacy at different wavelengths. Below we will discuss photochemical effects.

### 2.2. Dose

It is not difficult to find the dose dependence of bacterial viability from previous articles. The bacteria survival ratio decreases with the increase of the dose. Several studies [8,45,46] have shown that the photoinactivation dose curve is non-monoexponential and can be well described by the Hom model [47]. The Hom model of bacteria survival can be described as

(1) $$log\left(\frac{N}{N_{0}}\right)=ax^{b}$$

where *N* is the bacteria concentration after aBL exposure, $N_{0}$ is the initial concentration and *x* represents the dose. Further, *a* is a coefficient that is usually negative and *b* stands for the order of the exponential function. For *b* = 1, this model becomes log-linear, which also known as first-order kinetics. There are some other models that can be used to describe the inactivation curve after irradiation, such as the log-linear with shoulder and tail model and the Weibull model [34].

The dose curve of sessile bacteria has some differences in contrast to the bacteria in plankton. Ghate et al. showed that the inactivation of sessile *Listeria monocytogenes* at 410 nm began almost instantaneously and tailed off as the dose increased; however, it complete inactivation after 480 min illumination could not be achieved [14]. In contrast, the population of planktonic cells had a brief lag phase which lasted about 20 min, and then reduced below the detection limit at 84 min.

**Figure 1:**
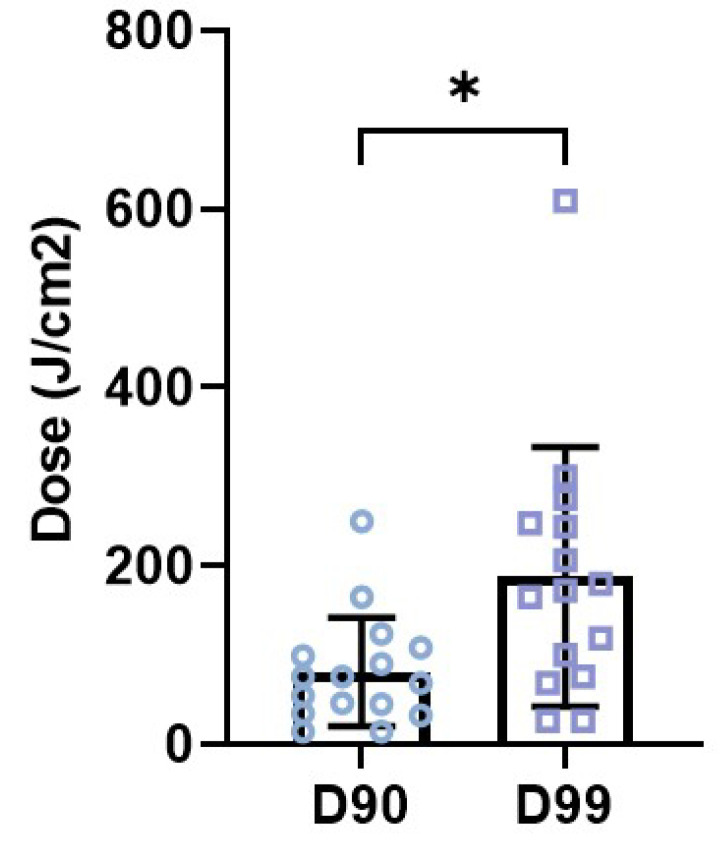
The recommended $D_{90}$ and $D_{99}$ of aBL treatment (at 400–420 nm) for *E. coli* from recent studies (* significant difference (p<0.05); circle: $D_{90}$ from different studies (see Table 1); box: $D_{99}$ from different studies (see Table 1)).

Generally, a higher dose leads to a greater antibacterial effect; however, time and economic cost still need to be considered in clinical and industrial settings. It is hard to give the exact value of $D_{90}$ and $D_{99}$ for various bacteria at various wavelengths. Taking *E. coli* as an example (which is widely studied in human health, food and environment [48] and is sensitive to blue light as a typical Gram-negative bacterium), we estimate that the dose for inactivation of 90% and 99% of *E. coli* (planktonic, in PBS) and the recommended $D_{90}$ and $D_{99}$ for aBL treatment (at 400–420 nm) were 81 and 188 J/cm^2^, respectively (see Figure 1).

### 2.3. Irradiance and Exposure Time

Although there is a view that the antibacterial effect of blue light mainly depends on the dose, some researchers have suggested that irradiance could affect aBL inactivation. A study showed the difference of photoinactivation on *Saccharomyces cerevisiae* caused by power intensity [49]. High irradiance (901.1 mW/cm^2^) showed the best inactivation, with a population decrease of 4.5 log CFU, while lower irradiance (225 mW/cm^2^ and 450 mW/cm^2^) induced a reduction lower than 3 log CFU at the same does. Sommers et al. placed foodborne pathogens on stainless steel coupons with 405 nm light exposure, and the log reductions ranged from 0.69 to 1.01 at an irradiance of 300 mW/cm^2^ versus 0.23 to 0.68 at 150 mW/cm^2^ [40]. Kotoku et al. showed some differences in survival ratios of *Porphyromonas gingivalis* at 100 mW/cm^2^ and 200 mW/cm^2^, namely, 0.46 and 0.121, respectively (405 nm, 2 J/cm^2^) [50]. Even though the antibacterial effect caused by irradiance may not be obvious as it caused by dose, it is still worth discussing. An extreme situation is that overly high irradiance will cause the waste of luminous energy, when the number of photons is much higher than the population of endogenous photosensitizers in bacteria. As for clinical and industrial application, increasing the irradiance properly helps to reduce time costs.

### 2.4. Frequency and Duty Cycle

**Figure 2:**
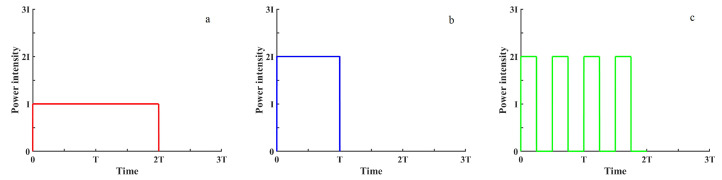
The different shapes of light in the same dose. Continuous wave light (**a**): $P_{L}=1$, t=2, $D_{c}=1$; (**b**): $P_{L}=2$, t=1, $D_{c}=1$ and pulsed light (**c**): $P_{L}=2$, t=2, $D_{c}=0.5$.

Compared with continuous wave light, pulsed light involves a change between light and dark (see Figure 2). When fitting dose and exposure time of aBL treatment with continuous wave light, it is inevitable to adjust the peak irradiance when changing the duty cycle in order to keep the same average irradiance. That is, it is hard to control only one variable in pulsed aBL experiments. Frequency (*f*) determines the speed of change, which varies from hundreds of milliseconds to several microseconds. The duty cycle ($D_{c}$) represents the proportion of light and dark time in a cycle ($T=\frac{1}{f}$). Light time and dark time are usually referred to as pulse width and pulse interval. We summarize some studies which used pulsed aBL in the following (see Table 3).

Gillespie et al. investigated the effects of duty cycle and frequency variation [39]. Dose and exposure time were constant in the experiment. The results show that the population of *Staphylococcus* aureus reduced to 0.04 similarly for all duty cycles (405 nm, $D_{c}$ = 0.25, 0.5, 0.75 or 1). Some fluctuations were observed, but there was no significant difference. Then, the duty cycle was fixed at 0.5 and the frequency was set as 100 Hz, 500 Hz, 1 kHz, 5 kHz, 10 kHz. There were significant differences between the 405 nm pulsed light exposure at 10 kHz and 1 kHz at the dose of 43.2 and 64.8 J/cm^2^. However, there was no significant difference among the populations after 86.4 J/cm^2^ illumination. The authors state that the average optical efficiency was increased by pulsed light compared with continuous exposure.

Enwemeka and his colleagues worked widely on the aBL treatment of *Propionibacterium acnes* and other bacteria with pulsed 450 nm light [44,51,52,53,54]. The results show that pulsed blue light suppressed the growth of *P. acnes* more than continuous wave (CW) blue light in vitro, and that treatment with 33% pulsed light gave the best result compared to a 20% pulsed wave or continuous wave. Furthermore, repeated irradiation at 3 h or 4 h intervals (coinciding with the replication cycle of *P. acnes*) enabled significant bacterial suppression even at lower irradiance (2 or 3 mW/cm^2^). Pulsed 450 nm light also suppressed the biofilm of *P. acnes* and MRSA.

It is well known that the peak irradiance cannot affect light penetration depth (which is dependent on wavelength and optical properties of media). Furthermore, the temperature of pulsed light treatment is the same as with CW light treatment, since the average irradiance is equal. We speculate that pulsed aBL might produce differences by impacting the photochemical efficiency or in other ways (e.g., photophysical effects), but more evidence is required.

### 2.5. Other Factors

To form treatment plans for clinical and industrial settings, it is necessary to investigate the blue light inactivation effect of bacteria in other liquids or on solid surfaces (see Table 2). Phosphate Buffered Saline (PBS) is a physiologically balanced buffer which does not support bacterial growth as a common media of experiments in vitro. In other media, the inactivation pattern of aBL can be very different from that in PBS, which is caused by factors such as bacteria growth phase, pH, and oxygen content. Since the nutrient media (e.g., milk, cantaloupe, TSB) is fit for bacterial propagation, the net inactivation could be a combined effect of aBL and bacterial growth. Some researchers have also investigated the differences in antimicrobial efficacy caused by initial concentration [38,55], growth phase [30] and temperature [11,45,56].

## 3. Damage and Protection Mechanisms of Antimicrobial Blue Light Therapy

**Figure 3:**
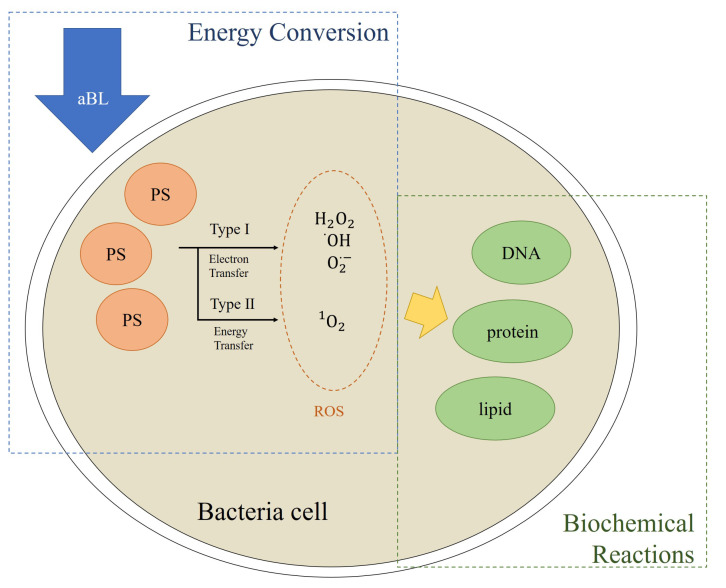
Two stages in the process of antimicrobial blue light therapy.

Based on the hypothesis of photosensitization and oxidative damage, there are two stages in the process of aBL therapy (Figure 3). In the first stage, endogenous photosensitizers absorb photons to become an excited state, and then reactions occur to produce ROS (e.g., hydrogen peroxide (H_2_O_2_), hydroxyl radicals(·OH), superoxide($O_{2}^{\cdot -}$), and singlet oxygen(O21)) [57]. In the second stage, oxidative damage occurs, and thus the quantity of viable bacteria decreases. Recently, some researchers proposed other damage mechanisms such as photophysical effects. Further, there are some protection mechanisms which help resist aBL stimulation. Thus, photoinactivation efficacy is affected by damage and protection mechanisms. In this part, we discuss the influence of aBL parameters on damage and protection mechanisms.

### 3.1. The Photochemical Effect of Endogenous Photosensitizers: Luminous Energy Conversion

#### 3.1.1. Endogenous Photosensitizers in Bacteria

Porphyrins (maximum absorption spectrum 405 nm) and flavins (maximum absorption spectrum 445 nm) are typical endogenous photosensitizers [8]. The metal-free porphyrins play an important role in antibacterial effects, as is already confirmed. Photochemically active monomer forms of porphyrins are coproporphyrin, protoporphyrin and uroporphyrin. The pyrrole subunits of porphyrins (e.g., coproporphyrin III and protoporphyrin IV) have conjugated π bonds which enable the strong absorption of blue light and effective conversion of photon energy [58]. The participation of flavins was discovered recently. Flavins contain double-bonded cyclic structures which act as acceptors of blue light and can be responsible for photoinactivation of bacteria.

#### 3.1.2. Energy Conversion in aBL Therapy

During the first stage, a part of luminous energy is converted to chemical energy (where the generation of ROS plays an essential role in photoinactivation), and the rest is consumed in other forms (e.g., thermal energy). The Type I reaction involves electron transfer while the Type II reaction involves energy transfer [59]. Hydrogen peroxide, hydroxyl radicals and superoxide anion radicals are generated in the Type I reaction [57,59], and the predominant Type II reactive oxygen species is singlet oxygen [57,60]. Although oxygen-independent photoreactions (Type III and Type IV) are reported in some studies, photodynamic action can reasonably expected to be oxygen-dependent at present [60].

The luminous energy (i.e., dose) can be described as

(2) $$E_{L}=P_{L}\times t\times D_{c}$$

where $E_{L}$ is the luminous energy per unit area emitted by light source, $P_{L}$ is the irradiance of light source, *t* is the exposure time and $D_{c}$ is the duty cycle of the light source. It is applicable to both continuous wave light and pulsed light. For continuous wave light, $D_{c}$ is equal to 1. For pulsed light, $D_{c}$ can be any value from 0 to 1. The chemical energy converted by luminous energy can be described as

(3) $$E_{C}=E_{L}\times \eta$$

where $E_{C}$ is the chemical energy and η is the conversion efficiency. The efficiency of energy conversion (η) can be described as

(4) $$\eta =\eta_{1}\times \eta_{2}$$

where $\eta_{1}$ is the ratio of photons received by photosensitizers to total emitted photons (in per unit area), and $\eta_{2}$ is the efficiency of producing ROS by photosensitizers after photons reception.

Assuming that the chemical energy is used for oxidative damage completely, it is easy to understand that dose plays a main role in chemical energy production, and other light parameters affect aBL therapy through energy conversion efficiency (η). The number of endogenous photosensitizers is $p_{0}$, and those in ground state and excited state are *p* and $p^{\prime }$ ($p+p^{\prime }=p_{0}$). In initial time (t=0), endogenous photosensitizers are all in the ground state, and one photosensitizer in ground state absorbs one photon to become an excited state [61]. There is time (Δt) for a photosensitizer becoming excited, joining the Type I or Type II reaction and coming back to the ground state.

For wavelength, it affects $\eta_{1}$ through the absorption spectrums of photosensitizers and impacts $\eta_{2}$ since the energy carried by photons at different wavelength is different. At a certain wavelength, the irradiance is proportional to the number of photons per unit time (*n*). We speculate that the irradiance affects energy conversion efficiency through $\eta_{1}$. If the number of photons is higher than the number of photosensitizers ($n>p_{0}$, t=0), every photosensitizer absorbs photons well but the rest of the photons are consumed. If the number of photons is lower than the number of photosensitizers ($n<p_{0}$, t=0), a part of the photosensitizers in the ground state are excited and the rest of the photosensitizers need to wait for more photons. When the first part of excited photosensitizers comes back to the ground state (t=Δt), if there is no photosensitizer which has not been excited (p=0), a part of the of photons are consumed; and if p>0, $\eta_{1}$ reaches the highest possible value in theory. Without considering exposure time, the low irradiance seems to reach high efficiency of energy conversion. However, in the clinical situation, over-low irradiance increases the time cost, and if irradiance is too high, it wastes luminous energy and accumulated temperature, which is not expected. Pulsed light has bright time ($t_{b}$) and dark time ($t_{d}$). Compared with CW light at the same peak irradiance, if $t_{b}<\Delta t$ and $t_{b}+t_{d}>\Delta t$, pulsed light improves the utilization efficiency of photons (especially when $n>p_{0}$).

As for $\eta_{2}$, it depends mainly on the type of photosensitizer. Some studies have shown the different quantum yield of singlet oxygen formation ($\phi_{\Delta }$) of uroporphyrin I and coproporphyrin III [62,63]. It is worth mentioning that $\eta_{2}$ can change during aBL treatment and the nature of this change is not clear at present. Many photosensitizers are consumed in the photochemical process, in fact [59]. Wu et al. found an obvious decrease in coproporphyrin levels in MRSA during aBL treatment [10].

#### 3.1.3. Intracellular Targets of ROS

The dose-dependent photoinactivation of bacteria after aBL treatment may be explained by the model of multiple targets. One of the major targets of ROS is the cell membrane. Studies have shown that aBL caused the depolarization of the membrane, the reduction of membrane integrity and alterations to membrane lipid profiles [10,11,64].

DNA is considered as another major target of ROS in bacteria. ROS can attack guanine bases and form oxidized derivatives, such as 8-hydroxy-deoxyguanosine (8-OHdG). Yoshida et al. confirmed that the generation of singlet oxygen caused higher 8-OHdG levels in blue-light-irradiated *P. gingivalis* cells [65]. The breakage of DNA only appeared during illumination, at which point the bacterial cell was no longer viable [66].

The cell wall of bacteria can be one of the targets. Giannelli et al. found the inactivation of lipopolysaccharide (LPS) in *E. coli* cells after 405 nm illumination [67]. In addition, Hu et al. observed an increased sensitivity of *Salmonella Typhimurium* to aBL exposure by disrupting the *rfaC* gene in *S. Typhimurium*, which caused the formation of truncated LPS and enhanced the permeability of the outer membrane [68].

### 3.2. Photophysical Effect of aBL

It seems that the phototoxicity of aBL on pathogenic bacteria is mainly attributed to the converted chemical energy (i.e., ROS). However, the possibility that photophysical effects play a role cannot be ruled out completely. Kim and Kang used mannitol as an ROS scavenger, and there was no significant difference on the damage of the cell membrane between the groups with and without mannitol after 395 nm and 405 nm light treatments [34]. In contrast, the integrity of the membrane varied in accordance with ROS when other wavelengths (415 and 425 nm) were used. The authors suggest that damage to cell structures might be caused by powerful photon strikes. Some studies on UV light showed physical destruction of bacteria cells [69,70], and there might be a similar effect in blue light. As a scavenger of hydroxyl radicals, mannitol cannot quench all kinds of reactive oxygen species. More evidence is required to find out whether the photophysical effect plays a role in aBL treatment.

### 3.3. Photoreceptors and Protection Mechanisms

Except endogenous photosensitizers, photoreceptors and some chemoreceptors in bacteria can also mediate the photosensing process in which blue light is used as a regulatory signal. There are three major photoreceptors (LOV, BLUF and PAS) in bacteria which have potential implications for aBL treatments [71]. Moreover, some research has shown evidence for the blue light sensing ability of *E. coli* chemoreceptors [72,73]. The blue light response of Aer was attributed to a PAS domain and it was not clear whether other chemoreceptors possess a chromophore-binding domain [71,74].

Bacteria can sense the rise of ROS levels and take some defensive measures. A main transcription factor involved in sensing oxidative stress is OxyR, which can detect the increased level of peroxide through thiol–disulfide exchange [57]. In *E. coli*, OxyR regulates several peroxide-detoxifying enzymes (e.g., KatG, AhpCF) and controls the import of manganese through the controlling gene *mntH* [75]. The gene *mntH* encodes MntH, which is the manganese importer. MntH can be strongly induced when cells are stressed by $H_{2}O_{2}$. Another established regulator of oxidative stress responses in *E. coli* are SoxR/SoxS systems which sense superoxide anions and partially protect cells from singlet oxygen [57,76]. In addition, bacteria may protect themselves from genotoxic stress via the SOS system to repair DNA in aBL treatment.

### 3.4. Resistance of Bacteria towards aBL

Due to the multiple targets of aBL therapy, the resistance of bacteria to aBL seems to be difficult to develop, and most studies have reached this consensus [77]. However, some researchers showed different findings. After being exposed to 15 cycles of sub-lethal 415 nm illumination, the cell wall of MRSA had significant thickening and the expression of peptidoglycan (PG) synthesis gene *glmS* was significantly upregulated [78]. It has been suggested that bacteria could develop resistance by changing their cell structure. Multiple sublethal aBL treatments could cause the genetic alterations which lead to the potential of tolerance development in representative Gram-negative bacteria [33]. More studies are needed to assess the possible resistance development of aBL. At the same time, more complex assessment and better terminology and methodology are also required [13].

## 4. Prospect

As a novel non-antibiotic antibacterial method, aBL therapy is proven to be effective in fighting the growth of bacteria. There are many parameters which impact the efficacy of aBL therapy. From the above, we suggest the use of shorter wavelength light (e.g., 405 nm) in aBL therapy. It is obvious that the dose plays an important role in photoinactivation, and we suggest the use of high irradiance or use pulsed aBL for shortening treatment time and improving the utilization efficiency of luminous energy. Considering the rise of temperature, the recommend irradiance is 50 mW/cm^2^; however, this may not the best irradiance. Future studies can explore higher peak irradiance through pulsed aBL. For clinical and industrial application, studies on different species and strains in different liquid or on different surfaces are excepted. At the same times, more studies are needed to assess the safety of aBL against host cells [16,79].

There are still some mysteries about the efficiency and mechanism of aBL therapy. The complexity of aBL treatment can be ascribed to the diverse parameters of aBL which impact antibacterial efficiency. First, the type and content of endogenous photosensitizers are different in various bacteria and related to the growth phase of bacteria. The photosensitizers are consumed gradually in the photochemical process, while it is unclear whether the synthesis of photosensitizers continues during aBL treatment. Besides, photoinactivation is the antagonism of damage mechanisms and protection mechanisms. Multi-targets in bacteria need to be destroyed by ROS to cause inactivation, and photophysical effects may also be involved. At the same time, some photoreceptors in bacteria which sense blue light can help to take some defensive measures, and resistance may be formed during aBL treatment. It is still worth investigating the influence of light parameters on photoinactivation and to evaluate the potential resistance of bacteria. More studies are needed to form treatment plans for clinical and industrial applications in the future.

## Figures and Tables

**Table 1:** Summary of continuous blue light inactivation of planktonic bacteria ($D_{c}=1$).

Species (Strains)	Initial Concentration	Wavelength (nm)	Power Intensity (mW/cm^2^)	D90, D99 (J/cm^2^)	References
*Escherichia coli* (ATCC 25922)	1.5 × 10^8^ CFU/mL	465	36.22	40	in saline solution [31]
*Escherichia coli* (ATCC 25922)	10^9^ CFU/mL	410	38.2	68.75, 206.25	[29]
	10^3^ CFU/mL	420	3	54, 75.6	
*Escherichia coli* (UTI 11380)	10^5^ CFU/mL			75.6, 119	[32]
	10^7^ CFU/mL			75.6, 172	
*Escherichia coli* (DSMZ 11250)	10^6^ CFU/mL	420	50	90, 180	[27]
		455		>90, 180	
		480		180	
*Escherichia coli* (ATCC 8739)	NS	405	50	99	[8]
		445		609	
*Escherichia coli* (ATCC 43895)	10^8^∼10^9^ CFU/mL	460–470	0.83	287	[11]
*Escherichia coli* (E2348/69)	10^9^ CFU/mL	410	38.2	45.9, 165	[29]
*Escherichia coli* (ST10)	10^9^ CFU/mL	410	38.2	34.375, 68.75	[29]
*Escherichia coli* (ST648)	10^9^ CFU/mL	410	38.2	165, 275	[29]
*Escherichia coli* (ST131)	10^9^ CFU/mL	410	38.2	123.75, 247.5	[29]
*Escherichia coli* (K-12)	10^7^∼10^8^ CFU/mL	415	NS	32.4	[33]
*Escherichia coli*	10^7^ CFU/mL	405	NS	250, 300	[12]
*Escherichia coli* (ATCC 35150 et al.)	10^9^ CFU/mL	395	13.8	18, 26	[34]
		405		14, 26	
		415		14, 26	
		425		18, 28	
		395		18, 30	
*Escherichia coli* (ATCC 35150 et al.)	10^9^ CFU/mL	405	13.8	18, 30	in juice [34]
		415		18, 30	
		425		20, 38	
*Escherichia coli* (NZRM 4519/81)	10^7^ CFU/mL	405	4.25	20	in LB [35]
*Escherichia coli* (NZRM 4566 et al.)	10^7^ CFU/mL	405	4.25	40	in LB [35]
*Escherichia coli* (NCTC 9001)	10^3^ CFU/mL	405	100	280, 320	in plasma [36]
	10^6^ CFU/mL			300, 360	[36]
MRSA (MRSA8325-4, MRSA252)	10^6^ CFU/mL	460	60	120, 240	[37]
MRSA (USA300)	5 × 10^6^ CFU/mL	470	25	55, 110	[38]
	7 × 10^6^ CFU/mL			165, 220	
*Staphylococcus aureus* (NCTC 4135)	10^3^ CFU/mL	405	16	70, 115	[39]
		420			
*Staphylococcus aureus* (DSMZ 799)	10^6^ CFU/mL	455	50	>180	[27]
		480			
*Staphylococcus aureus* (ATCC 25923)	NS	405	50	162	[8]
		445		606	
*Pseudomonas aeruginosa* (DSMZ 939)	10^6^ CFU/mL	420	50	NS, 60	[27]
		455		60, 90	
		480		90, 180	
*Listeria monocytogenes* (H7726)	NS	405	150	>180	chicken purge [40]
			300	180	

**Table 2:** Summary of continuous blue light inactivation of bacteria on solid surface ($D_{c}=1$).

Species (Strains)	Initial Concentration	Wavelength (nm)	Power Intensity (mW/cm^2^)	D90, D99 (J/cm^2^)	References
*Escherichia coli* (cocktail)	10^5^ CFU/mL	405	50	30	plastic steel
				108	stainless steel [15]
*Escherichia coli* (AF0006)	24-h old biofilms	405	60	>216	[41]
	48-h old biofilms			108	
*Escherichia coli* (CPE9606)	72-h old biofilms	405	60	54	[42]
			540	>162	
*Escherichia coli* (CPE7534)	72-h old biofilms	405	60	108	[42]
			540	>162	
*Listeria monocytogenes* (Δhly 10403 S)	10^5^ CFU/cm^2^	410	NS	18 min, 35 min	paperboard [14]
	10^7^ CFU/cm^2^			15 min, 30 min	

NS, not shown.

**Table 3:** Summary of pulsed blue light inactivation of planktonic bacteria ($D_{c}\in \left(0,1\right)$) ($D_{c}=1$).

Species (Strains)	Initial Concentration	Wavelength (nm)	Frequency (Hz), D_c_	P_max_, P_avg_ (mW/cm^2^)	D90, D99 (J/cm^2^)	References
*Staphylococcus aureus* (NCTC 4135)	10^3^ CFU/mL	405	100, 0.5	48, 24	86	[39]
			500, 0.5	48, 24	65, >86	
			1 k, 0.5	48, 24	43, 86	
			5 k, 0.5	48, 24	75	
			10 k, 0.5	48, 24	>86	
*Salmonella enterica Typhimurium* (ATCC13311)	10^9^ CFU/mL	455	100, 0.8	480, 391	436, >1047	dried food
					148, 279	pet food [43]
MRSA (USA300)	10^6^∼10^7^ CFU/mL	450	NS, 0.33	2	5.4 J	Biofilm [44]
*Propionibacterium acnes* (ATCC 6919)	10^6^∼10^7^ CFU/mL	450	NS, 0.33	2	4.5 J	Biofilm [44]

NS, not shown.

**Table 4:** The parameters of blue light.

Parameters	Symbol	Unit	Supplement
Wavelength	*λ*	nm	
Dose	*E_L_*	J/cm^2^	Luminous energy, energy intensity
Irradiance	*P_L_*	mW/cm^2^; W/cm^2^	Power intensity
Exposure time	*t*	s; min; h	
Duty cycle	*D_c_*		
Frequency	*f*	Hz	

## Data Availability

Data sharing is not applicable to this article as no new data were created or analyzed in this study.

## Abbreviations

The following abbreviations are used in this manuscript:

aBL	antimicrobial Blue Light
ROS	Reactive Oxygen Species
ARGs	Antibiotic Resistance Genes
CW	Continuous Wave
PBS	Phosphate Buffered Saline
TSB	Tryptone Soya Broth

## References

[1] Berendonk T.U., Manaia C.M., Merlin C., Fatta-Kassinos D., Cytryn E., Walsh F., Burgmann H., Sorum H., Norstrom M., Pons M.N. (2015). Tackling antibiotic resistance: The environmental framework. Nat. Rev. Microbiol..

[2] Singh R., Singh A.P., Kumar S., Giri B.S., Kim K.H. (2019). Antibiotic resistance in major rivers in the world: A systematic review on occurrence, emergence, and management strategies. J. Clean. Prod..

[3] Aminov R.I. (2010). A brief history of the antibiotic era: Lessons learned and challenges for the future. Front. Microbiol..

[4] Xu K.J., Song J., Zhao X.M. (2012). The drug cocktail network. BMC Syst. Biol..

[5] Dai T., Gupta A., Murray C.K., Vrahas M.S., Tegos G.P., Hamblin M.R. (2012). Blue light for infectious diseases: Propionibacterium acnes, Helicobacter pylori, and beyond?. Drug Resist. Updat..

[6] Wang Y.C., Wang Y., Wang Y.G., Murray C.K., Hamblin M.R., Hooper D.C., Dai T.H. (2017). Antimicrobial blue light inactivation of pathogenic microbes: State of the art. Drug Resist. Updat..

[7] Feuerstein O., Ginsburg I., Dayan E., Veler D., Weiss E.I. (2005). Mechanism of visible light phototoxicity on Porphyromonas gingivalis and Fusobacterium nucleatum. Photochem. Photobiol..

[8] Plavskii V.Y., Mikulich A.V., Tretyakova A.I., Leusenka I.A., Plavskaya L.G., Kazyuchits O.A., Dobysh I., Krasnenkova T.P. (2018). Porphyrins and flavins as endogenous acceptors of optical radiation of blue spectral region determining photoinactivation of microbial cells. J. Photochem. Photobiol. B-Biol..

[9] Hamblin M.R., Viveiros J., Yang C.M., Ahmadi A., Ganz R.A., Tolkoff M.J. (2005). Helicobacter pylori accumulates photoactive porphyrins and is killed by visible light. Antimicrob. Agents Chemother..

[10] Wu J., Chu Z., Ruan Z., Wang X., Dai T., Hu X. (2018). Changes of Intracellular Porphyrin, Reactive Oxygen Species, and Fatty Acids Profiles During Inactivation of Methicillin-Resistant Staphylococcus aureus by Antimicrobial Blue Light. Front. Physiol..

[11] Hyun J.E., Moon S.K., Lee S.Y. (2021). Antibacterial activity and mechanism of 460-470 nm light-emitting diodes against pathogenic bacteria and spoilage bacteria at different temperatures. Food Control.

[12] Leanse L.G., Harrington O.D., Fang Y., Ahmed I., Goh X.S., Dai T. Evaluating the potential for resistance development to antimicrobial blue light (at 405 nm) against Gram-negative bacteria: In vitro and in vivo studies. Proceedings of the Conference on Photonic Diagnosis and Treatment of Infections and Inflammatory Diseases II.

[13] Rapacka-Zdonczyk A., Wozniak A., Nakonieczna J., Grinholc M. (2021). Development of Antimicrobial Phototreatment Tolerance: Why the Methodology Matters. Int. J. Mol. Sci..

[14] Ghate V., Zelinger E., Shoyhet H., Hayouka Z. (2019). Inactivation of Listeria monocytogenes on paperboard, a food packaging material, using 410 nm light emitting diodes. Food Control.

[15] Wu S.Y., Hadi J., Brightwell G. (2022). Growth medium- and strain-dependent bactericidal efficacy of blue light against Shiga toxin-producing Escherichia coli on food-grade stainless steel and plastic. Food Microbiol..

[16] Leanse L.G., Dos Anjos C., Mushtaq S., Dai T. (2022). Antimicrobial blue light: A ’Magic Bullet’ for the 21st century and beyond?. Adv. Drug Deliv. Rev..

[17] Srimagal A., Ramesh T., Sahu J.K. (2016). Effect of light emitting diode treatment on inactivation of Escherichia coli in milk. Lwt-Food Sci. Technol..

[18] Jankowska K.I., Nagarkatti R., Acharyya N., Dahiya N., Stewart C.F., Macpherson R.W., Wilson M.P., Anderson J.G., MacGregor S.J., Maclean M. (2020). Complete Inactivation of Blood Borne Pathogen Trypanosoma cruzi in Stored Human Platelet Concentrates and Plasma Treated With 405 nm Violet-Blue Light. Front Med..

[19] Lu M., Dai T., Hu S., Zhang Q., Bhayana B., Wang L., Wu M.X. (2020). Antimicrobial blue light for decontamination of platelets during storage. J. Biophotonics.

[20] Wang Y., Wu X., Chen J., Amin R., Lu M., Bhayana B., Zhao J., Murray C.K., Hamblin M.R., Hooper D.C. (2016). Antimicrobial Blue Light Inactivation of Gram-Negative Pathogens in Biofilms: In Vitro and In Vivo Studies. J. Infect. Dis..

[21] Zhang Y., Zhu Y., Gupta A., Huang Y., Murray C.K., Vrahas M.S., Sherwood M.E., Baer D.G., Hamblin M.R., Dai T. (2014). Antimicrobial blue light therapy for multidrug-resistant Acinetobacter baumannii infection in a mouse burn model: Implications for prophylaxis and treatment of combat-related wound infections. J. Infect. Dis..

[22] Chui C., Aoki A., Takeuchi Y., Sasaki Y., Hiratsuka K., Abiko Y., Izumi Y. (2013). Antimicrobial effect of photodynamic therapy using high-power blue light-emitting diode and red-dye agent on Porphyromonas gingivalis. J. Periodontal Res..

[23] Genina E.A., Titorenko V.A., Belikov A.V., Bashkatov A.N., Tuchin V.V. (2015). Adjunctive dental therapy via tooth plaque reduction and gingivitis treatment by blue light-emitting diodes tooth brushing. J. Biomed. Opt..

[24] Soukos N.S., Stultz J., Abernethy A.D., Goodson J.M. (2015). Phototargeting human periodontal pathogens in vivo. Lasers Med. Sci..

[25] Lawrence C., Waechter S., Alsanius B.W. (2022). Blue Light Inhibits E. coli, but Decisive Parameters Remain Hidden in the Dark: Systematic Review and Meta-Analysis. Front. Microbiol..

[26] Tomb R.M., White T.A., Coia J.E., Anderson J.G., MacGregor S.J., Maclean M. (2018). Review of the Comparative Susceptibility of Microbial Species to Photoinactivation Using 380-480 nm Violet-Blue Light. Photochem. Photobiol..

[27] Plattfaut I., Demir E., Fuchs P.C., Schiefer J.L., Sturmer E.K., Bruning A.K.E., Oplander C. (2021). Characterization of Blue Light Treatment for Infected Wounds: Antibacterial Efficacy of 420, 455, and 480 nm Light-Emitting Diode Arrays Against Common Skin Pathogens Versus Blue Light-Induced Skin Cell Toxicity. Photobiomodulation Photomed. Laser Surg..

[28] Hessling M., Spellerberg B., Hoenes K. (2017). Photoinactivation of bacteria by endogenous photosensitizers and exposure to visible light of different wavelengths - a review on existing data. Fems Microbiol. Lett..

[29] dos Anjos C., Sabino C.P., Bueris V., Fernandes M.R., Pogliani F.C., Lincopan N., Sellera F.P. (2019). Antimicrobial blue light inactivation of international clones of multidrug-resistant Escherichia coli ST10, ST131 and ST648. Photodiagnosis Photodyn. Ther..

[30] Abana C.M., Brannon J.R., Ebbott R.A., Dunigan T.L., Guckes K.R., Fuseini H., Powers J., Rogers B.R., Hadjifrangiskou M. (2017). Characterization of blue light irradiation effects on pathogenic and nonpathogenic Escherichia coli. Microbiologyopen.

[31] Assuncao F.F.D., Nascimento E., Chaves L., da Silva A.M.H., Martinez R., Guirro R.R.D. (2022). Inhibition of bacterial growth through LED (light-emitting diode) 465 and 630 nm: In vitro. Lasers Med Sci..

[32] Vollmerhausen T.L., Conneely A., Bennett C., Wagner V.E., Victor J.C., O’Byrne C.P. (2017). Visible and UVA light as a potential means of preventing Escherichia coli biofilm formation in urine and on materials used in urethral catheters. J. Photochem. Photobiol. B-Biol..

[33] Rapacka-Zdonczyk A., Wozniak A., Kruszewska B., Waleron K., Grinholc M. (2021). Can Gram-Negative Bacteria Develop Resistance to Antimicrobial Blue Light Treatment?. Int. J. Mol. Sci..

[34] Kim D., Kang D.H. (2021). Efficacy of light-emitting diodes emitting 395, 405, 415, and 425 nm blue light for bacterial inactivation and the microbicidal mechanism. Food Res. Int..

[35] Wu S.Y., Ross C., Hadi J., Brightwell G. (2021). In vitro inactivation effect of blue light emitting diode (LED) on Shiga-toxin-producing Escherichia coli (STEC). Food Control.

[36] Stewart C.F., Tomb R.M., Ralston H.J., Armstrong J., Anderson J.G., MacGregor S.J., Atreya C.D., Maclean M. (2022). Violet-blue 405-nm Light-based Photoinactivation for Pathogen Reduction of Human Plasma Provides Broad Antibacterial Efficacy Without Visible Degradation of Plasma Proteins. Photochem. Photobiol..

[37] Yang P.G., Wang N., Wang C., Yao Y.F., Fu X.J., Yu W.R., Cai R., Yao M. (2017). 460 nm visible light irradiation eradicates MRSA via inducing prophage activation. J. Photochem. Photobiol. B-Biol..

[38] Bumah V.V., Masson-Meyers D.S., Enwemeka C.S. (2015). Blue 470nm light suppresses the growth of Salmonella enterica and methicillin-resistant Staphylococcus aureus (MRSA) in vitro. Lasers Surg. Med..

[39] Gillespie J.B., Maclean M., Given M.J., Wilson M.P., Judd M.D., Timoshkin I.V., MacGregor S.J. (2017). Efficacy of Pulsed 405-nm Light-Emitting Diodes for Antimicrobial Photodynamic Inactivation: Effects of Intensity, Frequency, and Duty Cycle. Photomed. Laser Surg..

[40] Sommers C., Gunther N.W., Sheen S. (2017). Inactivation of Salmonella spp., pathogenic Escherichia coli, Staphylococcus spp., or Listeria monocytogenes in chicken purge or skin using a 405-nm LED array. Food Microbiol..

[41] Ferrer-Espada R., Wang Y., Goh X.S., Dai T.H. (2020). Antimicrobial Blue Light Inactivation of Microbial Isolates in Biofilms. Lasers Surg. Med..

[42] Halstead F.D., Ahmed Z., Bishop J.R.B., Oppenheim B.A. (2019). The potential of visible blue light (405nm) as a novel decontamination strategy for carbapenemase-producing enterobacteriaceae (CPE). Antimicrob. Resist. Infect. Control.

[43] Prasad A., Ganzle M., Roopesh M.S. (2021). Antimicrobial activity and drying potential of high intensity blue light pulses (455 nm) emitted from LEDs. Food Res. Int..

[44] Bumah V.V., Masson-Meyers D.S., Enwemeka C.S. (2020). Pulsed 450 nm blue light suppresses MRSA and Propionibacteriurn acnes in planktonic cultures and bacterial biofilms. J. Photochem. Photobiol. B-Biol..

[45] Kumar A., Ghate V., Kim M.J., Zhou W.B., Khoo G.H., Yuk H.G. (2015). Kinetics of bacterial inactivation by 405 nm and 520 nm light emitting diodes and the role of endogenous coproporphyrin on bacterial susceptibility. J. Photochem. Photobiol. B-Biol..

[46] Kumar A., Ghate V., Kim M.J., Zhou W.B., Khoo G.H., Yuk H.G. (2017). Inactivation and changes in metabolic profile of selected foodborne bacteria by 460 nm LED illumination. Food Microbiol..

[47] Hom L.W. (1972). Kinetics of chlorine disinfection in an ecosystem. J. Sanit. Eng. Div. -Asce.

[48] Croxen M.A., Law R.J., Scholz R., Keeney K.M., Wlodarska M., Finlay B.B. (2013). Recent advances in understanding enteric pathogenic Escherichia coli. Clin. Microbiol. Rev..

[49] Lang E., Thery T., Peltier C., Colliau F., Adamuz J., Grangeteau C., Dupont S., Beney L. (2022). Ultra-high irradiance (UHI) blue light: Highlighting the potential of a novel LED-based device for short antifungal treatments of food contact surfaces. Appl. Microbiol. Biotechnol..

[50] Kotoku Y., Kato J., Akashi G., Hirai Y., Ishihara K. (2009). Bactericidal effect of a 405-nm diode laser on Porphyromonas gingivalis. Laser Phys. Lett..

[51] Bowman C., Bumah V.V., Niesman I.R., Cortez P., Enwemeka C.S. (2021). Structural membrane changes induced by pulsed blue light on methicillin-resistant Staphylococcus aureus (MRSA). J. Photochem. Photobiol. B-Biol..

[52] Bumah V.V., Masson-Meyers D.S., Tong W., Castel C., Enwemeka C.S. (2020). Optimizing the bactericidal effect of pulsed blue light on Propionibacterium acnes - A correlative fluorescence spectroscopy study. J. Photochem. Photobiol. B-Biol..

[53] Enwemeka C.S., Bumah V.V., Masson-Meyers D., Castel D., Castel C. Optimizing the bactericidal effect of pulsed blue light: A correlative fluorescence spectroscopy study. Proceedings of the Conference on Photonics in Dermatology and Plastic Surgery.

[54] Masson-Meyers D.S., Bumah V.V., Castel C., Castel D., Enwemeka C.S. (2020). Pulsed 450 nm blue light significantly inactivates Propionibacterium acnes more than continuous wave blue light. J. Photochem. Photobiol. B-Biol..

[55] Maclean M., MacGregor S.J., Anderson J.G., Woolsey G. (2009). Inactivation of Bacterial Pathogens following Exposure to Light from a 405-Nanometer Light-Emitting Diode Array. Appl. Environ. Microbiol..

[56] Price P.B., Sowers T. (2004). Temperature dependence of metabolic rates for microbial growth, maintenance, and survival. Proc. Natl. Acad. Sci. USA.

[57] Rapacka-Zdonczyk A., Wozniak A., Michalska K., Pieranski M., Ogonowska P., Grinholc M., Nakonieczna J. (2021). Factors Determining the Susceptibility of Bacteria to Antibacterial Photodynamic Inactivation. Front. Med..

[58] Bumah V.V., Morrow B.N., Cortez P.M., Bowman C.R., Rojas P., Masson-Meyers D.S., Suprapto J., Tong W.G., Enwemeka C.S. (2020). The importance of porphyrins in blue light suppression of Streptococcus agalactiae. J. Photochem. Photobiol. B-Biol..

[59] Baptista M.S., Cadet J., Greer A., Thomas A.H. (2021). Photosensitization Reactions of Biomolecules: Definition, Targets and Mechanisms. Photochem. Photobiol..

[60] Baptista M.S., Cadet J., Di Mascio P., Ghogare A.A., Greer A., Hamblin M.R., Lorente C., Nunez S.C., Ribeiro M.S., Thomas A.H. (2017). Type I and Type II Photosensitized Oxidation Reactions: Guidelines and Mechanistic Pathways. Photochem. Photobiol..

[61] Cieplik F., Pummer A., Regensburger J., Hiller K.A., Spath A., Tabenski L., Buchalla W., Maisch T. (2015). The impact of absorbed photons on antimicrobial photodynamic efficacy. Front. Microbiol..

[62] Blum A., Grossweiner L.I. (1985). Singlet oxygen generation by hematoporphyrin-ix, uroporphyrin-i and hematoporphyrin derivative at 546 nm in phosphate buffer and in the presence of egg phosphatidylcholine liposomes. Photochem. Photobiol..

[63] Lambert C.R., Reddi E., Spikes J.D., Rodgers M.A.J., Jori G. (1986). The effects of porphyrin structure and aggregation state on photosensitized processes in aqueous and micellar media. Photochem. Photobiol..

[64] McKenzie K., Maclean M., Grant M.H., Ramakrishnan P., MacGregor S.J., Anderson J.G. (2016). The effects of 405 nm light on bacterial membrane integrity determined by salt and bile tolerance assays, leakage of UV-absorbing material and SYTOX green labelling. Microbiology.

[65] Yoshida A., Sasaki H., Toyama T., Araki M., Fujioka J., Tsukiyama K., Hamada N., Yoshino F. (2017). Antimicrobial effect of blue light using Porphyromonas gingivalis pigment. Sci. Rep..

[66] Nitzan Y., Ashkenazi H. (2001). Photoinactivation of Acinetobacter baumannii and Escherichia coli B by a cationic hydrophilic porphyrin at various light wavelengths. Curr. Microbiol..

[67] Giannelli M., Landini G., Materassi F., Chellini F., Antonelli A., Tani A., Nosi D., Zecchi-Orlandini S., Rossolini G.M., Bani D. (2017). Effects of photodynamic laser and violet-blue led irradiation on Staphylococcus aureus biofilm and Escherichia coli lipopolysaccharide attached to moderately rough titanium surface: In vitro study. Lasers Med. Sci..

[68] Hu X.Q., Zhang X.J., Luo S.H., Wu J.X., Sun X.Y., Liu M.M., Wang X.Y., Wang X.H. (2021). Enhanced Sensitivity of Salmonella to Antimicrobial Blue Light Caused by Inactivating rfaC Gene Involved in Lipopolysaccharide Biosynthesis. Foodborne Pathog. Dis..

[69] Ramos-Villarroel A.Y., Aron-Maftei N., Martin-Belloso O., Soliva-Fortuny R. (2012). The role of pulsed light spectral distribution in the inactivation of Escherichia coli and Listeria innocua on fresh-cut mushrooms. Food Control.

[70] Xiao Y., Chu X.N., He M., Liu X.C., Hu J.Y. (2018). Impact of UVA pre-radiation on UVC disinfection performance: Inactivation, repair and mechanism study. Water Res..

[71] Hadi J., Wu S., Soni A., Gardner A., Brightwell G. (2021). Genetic Factors Affect the Survival and Behaviors of Selected Bacteria during Antimicrobial Blue Light Treatment. Int. J. Mol. Sci..

[72] Perlova T., Gruebele M., Chemla Y.R. (2019). Blue Light Is a Universal Signal for Escherichia coli Chemoreceptors. J. Bacteriol..

[73] Wright S., Walia B., Parkinson J.S., Khan S. (2006). Differential activation of Escherichia coli chemoreceptors by blue-light stimuli. J. Bacteriol..

[74] Taylor B.L. (2007). Aer on the inside looking out: Paradigm for a PAS-HAMP role in sensing oxygen, redox and energy. Mol. Microbiol..

[75] Cornelis P., Wei Q., Andrews S.C., Vinckx T. (2011). Iron homeostasis and management of oxidative stress response in bacteria. Metallomics.

[76] Anjem A., Varghese S., Imlay J.A. (2009). Manganese import is a key element of the OxyR response to hydrogen peroxide in Escherichia coli. Mol. Microbiol..

[77] Marasini S., Leanse L.G., Dai T.H. (2021). Can microorganisms develop resistance against light based anti-infective agents?. Adv. Drug Deliv. Rev..

[78] Luo S.H., Yang X., Wu S.Y., Li Y.B., Wu J.X., Liu M.M., Liu Z.J., Yu K.Y., Wang X.Y., Dai T.H. (2022). Understanding a defensive response of methicillin-resistant Staphylococcus aureus after exposure to multiple cycles of sub-lethal blue light. Fems Microbiol. Lett..

[79] Jana S., Heaven M.R., Dahiya N., Stewart C., Anderson J., MacGregor S., Maclean M., Alayash A.I., Atreya C. (2023). Antimicrobial 405 nm violet-blue light treatment of ex vivo human platelets leads to mitochondrial metabolic reprogramming and potential alteration of Phospho-proteome. J. Photochem. Photobiol. B.

